# Belief in false information regarding the COVID-19 pandemic and a tendency to conspiracy thinking as factors that may exacerbate symptoms of anxiety and depression among Polish citizens

**DOI:** 10.1192/j.eurpsy.2022.233

**Published:** 2022-09-01

**Authors:** P. Dębski, M. Piegza, N. Kapuśniak, A. Boroń, M. Dębska, P. Gorczyca

**Affiliations:** 1 Medical University of Silesia, Department Of Psychiatry, Tarnowskie Gory, Poland; 2 The Jerzy Kukuczka Academy of Physical Education in Katowice, Institute Of Sport Sciences, Katowice, Poland

**Keywords:** Covid-19, conspiracy thinking, Depression, Anxiety

## Abstract

**Introduction:**

The COVID-19 pandemic has become the subject of intense discussion on social media platforms. Fake news and conspiracy theories about the SARS-CoV-2 virus, in particular its origin, spread, impact on health and prevention, have become especially popular. The social crisis triggered by the COVID-19 pandemic is associated with a growing tendency to believe in conspiracy theories, which in turn may contribute to an increase in anxiety tension and thus deteriorate the psychological health of citizens.

**Objectives:**

The aim of the study was to determine the relationships between the tendency to believe in false information about the COVID-19 pandemic and the severity of symptoms of anxiety and depression among the surveyed Polish citizens.

**Methods:**

The study included 700 Polish people aged 24.7±6.34 years. We used questionnaires such as: COVID-19 Conspiratorial Beliefs Scale to measure the level of belief in false information regarding the COVID-19 pandemic, Generic Conspiracist Beliefs Scale to measure tendencies to believe in conspiracy theories, and Hospital Anxiety and Depression Scale.

**Results:**

Belief in false information about the COVID-19 pandemic may be associated with a slight increase in the severity of both anxiety symptoms (b=0.044; p=0.021) and depression (b=0.048; p=0.004). A factor known as belief in the criminal activity of government organizations may also contribute to predicting the increase in the severity of symptoms of anxiety (b=0.172; p=0.001) and depression (b=0.169; p=0.000) during the COVID-19 pandemic.

**Conclusions:**

Belief in false information about the COVID-19 pandemic, as well as belief in general conspiracy theories, can contribute to the psychological deterioration of citizens during the COVID-19 pandemic.

**Disclosure:**

No significant relationships.

